# Seasonal Influence on the Bioactive Profile of Essential Oil from Azorean *Cryptomeria japonica* Foliage: In Vitro and In Silico Studies

**DOI:** 10.3390/molecules31142532

**Published:** 2026-07-21

**Authors:** Tânia Rodrigues, Ana Lima, Jorge Frias, Lua Palmeira, Filipe Arruda, Alexandre Janeiro, José Baptista, Elisabete Lima

**Affiliations:** 1 Institute of Agricultural and Environmental Research and Technology (IITAA), University of the Azores, 9700-042 Angra do Heroísmo, Portugal; tanyamsrod@gmail.com (T.R.); filipearruda1995@hotmail.com (F.A.); alexandre.p.janeiro@uac.pt (A.J.); jose.ab.baptista@uac.pt (J.B.); 2 Department of Biology (DB), Faculty of Science and Technology, University of the Azores, 9500-321 Ponta Delgada, Portugal; jorge.mv.frias@uac.pt; 3 Department of Physics, Chemistry and Engineering (DCFQE), Faculty of Science and Technology, University of the Azores, 9500-321 Ponta Delgada, Portugal; lua.palmeira@outlook.com; 4 Biotechnology Centre of Azores (CBA), University of the Azores, 9700-042 Angra do Heroísmo, Portugal

**Keywords:** *Cryptomeria japonica*, biomass valorization, essential oil, seasonal variation, antibacterial, antioxidant, anti-inflammatory, kaur-16-ene, molecular docking, circular bioeconomy

## Abstract

Driven by the growing demand for quality assurance within the essential oil (EO) industry, this study builds upon prior seasonal chemical and anticholinergic characterizations of Azorean *Cryptomeria japonica* foliage (Az–CJF) EO by presently evaluating the seasonal variations in its antibacterial, antioxidant, and anti-inflammatory activities. Autumn (Aut–EO) and spring (Spr–EO) samples exhibited a uniform, targeted antibacterial profile exclusively against Gram-positive bacteria, with a MIC of 5.0 mg/mL for *Micrococcus luteus* and ≥10.0 mg/mL for *Bacillus licheniformis*, *B. subtilis*, and *Staphylococcus aureus*. Antioxidant capacities also remained seasonally consistent within each of the three individual assays, namely the DPPH, ABTS, and β-carotene bleaching assays (EC_50_ ≤ 10.5, ≤ 6.0, and ≤0.3 mg/mL, respectively), suggesting a stable potential for lipid peroxidation inhibition. In addition, both EOs protected against bovine serum albumin denaturation (84–98%) with no statistically significant differences, generally outperforming diclofenac sodium (70–87%). Notably, anti-inflammatory activity via COX pathways proved to be seasonally dependent: Spr–EO inhibited COX-1 and COX-2 more significantly than Aut–EO, with both EOs showing COX-1 selectivity (IC_50_ of 284 vs. 560 µg/mL). Although less potent than diclofenac sodium (COX IC_50_ < 0.2 µg/mL), the superior activity of Spr–EO was supported by molecular docking, suggesting this enhanced effect is driven by higher contents of kaur-16-ene and oxygenated sesquiterpenes (such as α- and β-eudesmol). These bioactivities provide baseline parameters for batch standardization. By merging stable core properties with seasonal anti-inflammatory profiles, Az–CJF EO shows promise as a natural multi-target-directed ligand (MTDL) for therapeutic applications within a circular bioeconomy.

## 1. Introduction

Nowadays, there is a renewed interest in utilizing bioactive natural products, particularly plant secondary metabolites (PSMs), for diverse commercial purposes. Indeed, beyond their crucial ecological roles, particularly in plant resistance and adaptation, PSMs offer vast potential for drug discovery and green plant protection [1,2,3,4,5].

Among these metabolites, essential oils (EOs) are particularly valued for their broad-spectrum bioactivities, which enable them to act as multi-target-directed ligands (MTDLs) owing to their complex, multi-component chemical profile. Furthermore, they are generally recognized as eco-friendly products that can offer lower toxicity and superior safety profiles compared to their synthetic counterparts. Essential oil components (EOCs) are predominantly terpenes/terpenoids (the largest, most chemically and functionally diverse class of PSMs) and phenylpropanoids [3,6]. However, the chemical profile of an EO, and consequently its specific commercial applications and market value, is determined by a myriad of endogenous and/or exogenous factors, which can even lead to distinct EO chemotypes within the same plant species. These factors include the plant species, surrounding abiotic and biotic environmental conditions, phenology, and specific plant organs used [7]. While extraction methods also influence the final profile, the International Organization for Standardization (ISO) strictly defines EOs as products obtained through hydrodistillation (HD), steam distillation, dry distillation, or cold pressing (for citrus peels) [8].

Commercially important EOs are found across approximately 60 plant families, including the coniferous Cupressaceae [9]. Recent comprehensive screenings within this family have shown that EOs from woody plant resources exhibit marked chemical diversity and seasonal variations in their active chemotypes, serving as high-value, sustainable assets for natural antibacterial development [10]. The Cupressaceae family includes the genus *Cryptomeria*, consisting of a single species: *Cryptomeria japonica* (Thunb. ex L. f.) D. Don, the target plant in the present study. Native to Japan, this aromatic tree is a primary plantation species in both the Far East and the Azores archipelago (Portugal). While primarily valued as a building material, *C. japonica* also serves as a source of natural products in traditional Asian medicine, specifically for the treatment of coughs, ulcers, and liver-related pathologies [11,12].

Like most coniferous trees, *C. japonica* possesses evergreen foliage (CJF), which is the specific plant part used in this study. The leaves are needle-like and arranged in a compact spiral structure. As a monoecious species, each *C. japonica* individual bears both male and female reproductive organs. The male cones disperse pollen during the spring, while the female cones house the developing seeds until they reach maturity in autumn [13].

Although CJF biomass yields a valuable terpene-rich EO, this sustainable resource remains relatively underutilized for medical and technological applications. In fact, a critical review by Lima et al. [12] highlighted the diverse chemical compositions and bioactivities of CJF EO across different geographical origins. That research [12] has classified the CJF EOs into three distinct chemotypes, based on their major EOCs: (i) *ent*-kaurene chemotype (prevalent in Korea and Taiwan); (ii) elemol plus *ent*-kaurene chemotype (common in Japan, China, and Nepal) and (iii) α-pinene chemotype (frequent in the Azores, Corsica, and Japan). Even though individual bioactive potencies may differ, all these CJF EO chemotypes are consistently recognized for their multi-target antimicrobial and pesticidal (e.g., termiticidal and acaricidal) efficacy. Other important CJF EO bioactivities, although more scarcely reported, include antioxidant, anti-inflammatory, cancer chemopreventive, and neuropharmacological properties. Consequently, *C. japonica* EO and/or its individual EOCs hold great promise for innovative applications in healthcare, food preservation, agriculture, and the environmental sector [12,14,15,16,17].

In the Azores archipelago, *C. japonica* occupies nearly 60% of the total wood-producing forest area, with the largest plantations located on São Miguel Island [18]. Forestry operations and wood processing generate substantial amounts of biomass residues [19], particularly CJF (referred to hereafter as Az–CJF). This residue is used locally for EO production, providing an economically and environmentally sustainable strategy that avoids land-use competition.

As global demand for EOs grows [20], the industry requires rigorous quality assurance to guarantee standardized product performance. Consequently, it is important to investigate the seasonal variations in the chemical composition and bioactivity of Az–CJF EOs.

In this context, recent work by the research group [21] demonstrated that EOs hydrodistilled from Az–CJF, collected in autumn (Aut–EO) and spring (Spr–EO), displayed no toxicity in the brine shrimp lethality assay and exhibited consistent anti-butyrylcholinesterase activity, despite seasonal variations in their quantitative composition. Building upon these findings, the two seasonal EOs were investigated herein regarding three primary in vitro bioactivities: (i) antibacterial effects against seven spoilage-forming and/or pathogenic bacteria, determining growth inhibition zones (GIZs) and minimum inhibitory concentrations (MIC) via the disc diffusion method (DDM) and broth microdilution method (BMM), respectively; (ii) antioxidant potential, estimated through free radical scavenging activity (FRSA), using 2,2-diphenyl-1-picrylhydrazyl (DPPH) and 2,2′-azino-bis(3-ethylbenzothiazoline-6-sulfonic acid) (ABTS) radicals, alongside the β-carotene-linoleic acid bleaching activity (BCBA) emulsion system; and (iii) anti-inflammatory potential, assessed via bovine serum albumin (BSA) denaturation inhibition, and cyclooxygenase (COX-1/COX-2) inhibition assays. In addition, molecular docking was employed to explore the potential interactions between EOCs and the catalytic pockets of both COX isoforms. Consequently, this study establishes the first comprehensive seasonal baseline for the antibacterial, antioxidant, and in vitro/in silico anti-inflammatory profiles of Az–CJF EO, addressing a critical gap in the pharmacological valorization of this conifer resource.

## 2. Results and Discussion

### 2.1. Yield and Chemical Composition of EOs Obtained via HD from Fresh Az–CJF Samples Collected in Autumn and Spring

**Figure 1:**
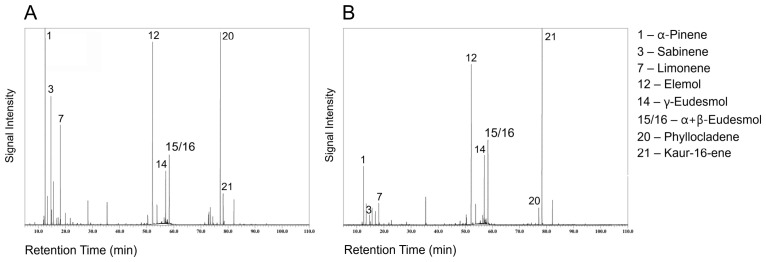
GC–MS total ion current (TIC) chromatogram of the essential oil obtained from Azorean *Cryptomeria japonica* foliage harvested in autumn (**A**) and spring (**B**) (adapted from Rodrigues et al. [21]). Major components (>5% total peak area) are assigned in the chromatograms.

The Az–CJF samples under study, representing two distinct developmental stages, specifically seed dispersal (autumn) and pollen release (spring), were previously found to be a good source of EO (>10 mL/kg on a dry matter basis) [21], with the harvesting season showing no statistically significant influence on the Az–CJF EO yield (Table 1). In terms of chemical characterization, a total of 104 EOCs were established across the investigated Az–CJF EOs [21]. Among these components, 91 were simultaneously detected in both the Aut–EO and Spr–EO samples, spanning six distinct chemical classes: monoterpene hydrocarbons (MH), oxygenated monoterpenes (OM), sesquiterpene hydrocarbons (SH), oxygenated sesquiterpenes (OS), diterpene hydrocarbons (DH), and oxygenated diterpenes (OD). Qualitatively, the profiles of both seasonal EOs remained remarkably close, sharing an identical core of 78 EOCs. Conversely, the relative percentages of several individual components fluctuated significantly between the collection periods [21], a variation consistent with the metabolic synchronization typically observed between photosynthesis and plant terpene pathways. The twenty-two major EOCs (≥1%), alongside the cumulative values for each specialized chemical class, are detailed in Table 1, while the corresponding gas chromatography–mass spectrometry (GC–MS) total ion current (TIC) chromatograms for the Aut–EO and Spr–EO samples are depicted in Figure 1A,B, respectively.

A comparison of the chemical profiles revealed that Aut–EO (November 2022) is significantly richer in total terpenes, particularly MH (35% vs. 16%), mainly α-pinene (**1**), sabinene (**3**), and limonene (**7**). Conversely, Spr–EO (April 2023) contains a higher proportion of total terpenoids, particularly OS (45% vs. 31%), mainly elemol (**12**), α+β-eudesmol (**15** plus **16**), and γ-eudesmol (**14**). These variations resulted in distinct terpene/terpenoid ratios of 1.7 for Aut–EO and 0.9 for Spr–EO. In addition, both samples were rich in DH (26% vs. 29%), with phyllocladene (**20**) predominating in Aut–EO and kaur-16-ene (**21**) in Spr–EO, which constitute their respective major EOCs (Table 1 and Figure 1).

Characterizing these seasonal shifts is crucial for interpreting the biological activities of the studied EOs. In fact, the bioactive efficacy of EOs depends heavily on the chemical nature of their EOCs, as well as on the complex synergistic, additive, or antagonistic interactions between major and minor components [22].

### 2.2. In Vitro Biological Activities of EOs Obtained via HD from Fresh Az–CJF Samples Collected in Autumn and Spring

#### 2.2.1. Antibacterial Activity (Growth Inhibitory Effects)

The search for PSMs, such as EOs, with antibacterial properties aims to address the rising resistance of pathogenic bacteria to current synthetic antibiotics. Indeed, as natural MTDLs, EOs interact with multiple cellular targets simultaneously, significantly reducing the likelihood of bacterial resistance [22,23]. In this context, the seasonal effect on the antibacterial activity of the studied Az–CJF EOs, alongside the antibiotic kanamycin as the positive control, was evaluated using the DDM and BMM. The results, expressed as GIZ and MIC values, are summarized in Table 2.

The seasonal EOs’ GIZ results revealed no statistically significant differences between the two samples across any of the selected strains. Both samples displayed activity exclusively against Gram-positive bacteria, with GIZ values of 6.1–7.8 mm for Aut–EO and 6.1–8.1 mm for Spr–EO, while remaining inactive against all Gram-negative bacteria. Within the Gram-positive bacteria, the inhibitory activity decreased in the following order: *B. licheniformis* > *B. subtilis* ≅ *S. aureus* > *M. luteus*. However, analysis of the EOs’ MIC results revealed that *M. luteus* emerged as the most susceptible strain to both EOs, exhibiting an identical MIC value of 5.0 mg/mL for both studied seasons, compared to the MIC ≥ 10.0 mg/mL observed for the other tested bacteria. This specific susceptibility in the BMM contrasts with the lower diffusion observed in the DDM. This discrepancy is standard and is attributed to the hydrophobicity and viscosity of undiluted EOs, which limit their diffusion in agar but are successfully overcome in the BMM by utilizing dimethyl sulfoxide (DMSO) as a solubilizing agent [24].

Regarding the available literature data on the antibacterial activity of Az–CJF’s EOCs [10,15,25,26,27], it can be pointed out that Az–CJF EO, from both studied seasons, is a raw material containing several active EOCs, including α-pinene (**1**), sabinene (**3**), β-pinene (**4**), limonene (**7**), γ-terpinene (**8**), terpinen-4-ol (**9**), bornyl acetate (**10**), eudesmol isomers (**14**–**16**), phyllocladene (**20**), and kaur-16-ene (**21**) (Table 1). As is well established, within the terpene/terpenoid class, oxygenated terpenoids typically exhibit higher antibacterial activity than their terpene hydrocarbon counterparts [10,27].

Under the applied experimental criteria, the present data characterize Az–CJF EO as a weak antibacterial agent compared to the positive control. However, it still exerts measurable, seasonally consistent growth inhibition against a broad range of pathogenic Gram-positive bacteria, demonstrating its baseline potential as a conifer defense resource. In fact, conifer EOs typically exhibit antibacterial properties because these plants produce terpene- and terpenoid-rich oleoresins, which serve as signature components in plant defense against biotic threats [28]. Specifically, these conifer EOs combat bacteria primarily by compromising membrane structures and functions. This toxicity is driven by the high hydrophobicity of hydrocarbon molecules, particularly cyclic MH [29,30]. The varying antibacterial effectiveness of conifer EOs likely stems from different diffusion rates of active EOCs through the cell wall and into membrane phospholipids [31], a phenomenon that may be partly explained by their distinct physicochemical properties. Furthermore, the present results confirmed the pattern already highlighted in *C. japonica* EO action as an antibacterial agent with greater efficacy against Gram-positive than Gram-negative bacteria [31,32].

In this context, Inoue et al. [26] suggested that the antibacterial activity of Japanese CJF EO is partly driven by α-pinene (**1**), alongside minor EOCs that act as synergistic enhancers. This evolutionary, multi-component defense strategy is further evidenced within the Cupressaceae family. Recently, Huang et al. [10] reported that seasonal fluctuations in major EOCs, including α-pinene (**1**) and sabinene (**3**), did not fundamentally alter the baseline qualitative antibacterial spectrum of 11 related species, mirroring the seasonal stability observed in the Az–CJF EO.

Crucially, Huang et al. [10] demonstrated that efficacy against Gram-negative bacteria requires a significant concentration of moderately polar OM, such as terpinen-4-ol (**9**), which can efficiently penetrate the lipopolysaccharide (LPS) outer membrane barrier. In contrast, non-polar terpenes, such as *α*-pinene (**1**), sabinene (**3**), and *γ*-terpinene (**8**), are often hindered by this LPS layer, while bulky or highly polar oxygenated derivatives, such as bornyl acetate (**10**), diffuse too slowly. The analyzed Az–CJF EO samples were characterized by a very low total OM content (4.5% for Aut–EO vs. 3.9% for Spr–EO), with terpinen-4-ol (**9**) reaching only 2.0% in Aut–EO and bornyl acetate (**10**) 2.5% in Spr–EO (Table 1). Conversely, both seasonal samples were heavily dominated by OS (30.7% for Aut–EO vs. 44.6% for Spr–EO). Consequently, the volatile profile is dominated by high-molecular-weight OS (such as elemol and eudesmol isomers) rather than the small, highly permeable OM identified by Huang et al. [10] as Gram-negative permeabilizers. These bulky EOCs, despite their structural complexity, lack the specific size and polarity required to overcome the Gram-negative outer membrane barrier, thereby restricting the EO’s bioactivity to Gram-positive strains.

In agreement with these findings, the present results confirm that while quantitative chemical shifts exist between the autumn and spring harvests of Az–CJF, such as the variation in the terpene/terpenoid ratio (1.7 for Aut–EO vs. 0.9 for Spr–EO), the core antibacterial profile remains remarkably stable. From an applied perspective, this chemical complexity highlights the advantage of utilizing the whole, crude Az–CJF EO rather than isolated fractions or individual EOCs. As emphasized by recent data-driven models [10], reductionist approaches frequently fail to capture the vital cumulative or synergistic interactions among multiple EOCs, which are essential to achieve effective multi-target interactions while maintaining acceptable toxicological profiles in practical applications.

#### 2.2.2. Antioxidant Activity (DPPH/ABTS–FRSA and BCBA)

The antioxidant activity of EOs is a biological property of increasing interest, especially due to concerns regarding the potential toxic side effects of most of the synthetic antioxidants used in the medical, cosmetic, and food sectors. In this context, the seasonal effect on the antioxidant potential of the studied Az–CJF EOs, alongside the reference antioxidant gallic acid as the positive control, was evaluated using different methods to better understand the antioxidant mechanisms displayed by the EO samples. The results, expressed as half-maximal effective concentration (EC_50_) values, are summarized in Table 3.

As shown in Table 3, no statistically significant differences were found between the Aut–EO and Spr–EO within each of the three individual assays, demonstrating a consistent trend with the seasonally stable antibacterial activity results.

The EC_50_ values for Aut–EO and Spr–EO, respectively, were as follows: 0.2 and 0.3 mg/mL for the BCBA; 5.6 and 6.0 mg/mL for the ABTS–FRSA; and 10.5 and 8.2 mg/mL for the DPPH–FRSA. A significant correlation was found among the methods used for antioxidant activity determination between both EO samples, as assessed by Pearson’s correlation coefficients. Moderate correlations (0.3 < ∣*r*∣ < 0.7) were observed between DPPH–FRSA and either ABTS–FRSA or BCBA (*r* = 0.533 and *r* = 0.611, respectively), while a strong correlation (∣*r*∣ > 0.7) was found between ABTS–FRSA and BCBA (*r* = 0.783).

Due to the demonstrated antioxidant effectiveness in a lipophilic environment, the studied seasonal EOs may play an important role in protecting against or delaying lipid oxidation. In fact, it appears to be a general pattern that EOs containing monoterpenes, monoterpenoids, and/or sesquiterpenes (both hydrocarbons and oxygenated derivatives) exhibit a higher antioxidant potential, particularly those richest in oxygenated compounds [33,34]. In the specific case of the Az–CJF EO, the marked enrichment in OS during spring (44.6% in Spr–EO vs. 30.7% in Aut–EO) likely compensated for the concurrent reduction in MH (15.8% in Spr–EO vs. 35.0% in Aut–EO) (Table 1). This chemical trade-off successfully maintained a steady-state antioxidant baseline across seasons, representing a functional homeostatic feature that is highly desirable for future industrial batch standardization.

The significantly higher activity displayed by the seasonal EOs in the BCBA assay, compared to the FRSA tests, may be associated with the higher specificity of the former for lipophilic compounds. In this assay, the reaction mechanism involves the formation of a radical adduct [terpene–OOR^●^] with the participation of terpenes possessing double bonds, including α-pinene (**1**), sabinene (**3**), and γ-terpinene (**8**), which are well-represented in the Az–CJF EO (Table 1). Conversely, for DPPH and ABTS radicals, the mechanism (particularly in methanol) frequently involves a mixed hydrogen atom transfer (HAT)/single electron transfer (SET) pathway [35], explaining the higher EC_50_ values obtained due to the absence of strong phenolic hydrogen- or electron-donors in this conifer EO. Nevertheless, both the Aut–EO and Spr–EO samples were characterized by high levels of elemol (**12**; 16.0% vs. 20.8%) and eudesmol isomers (**14**–**16**; 11.7% vs. 18.6%), which are documented as active agents in these pathways, especially γ-eudesmol (**14**; 3.9% vs. 6.0%). These EOCs, alongside the presence of nezukol (**22**; 1.6% vs. 2.7%) (Table 1), likely contribute to the FRSA properties of the Az–CJF EO [32].

Concerning the DPPH/ABTS–FRSA assays, both EOs exhibited a slightly higher antioxidant capacity in the ABTS than in the DPPH (1.9- and 1.4-fold greater for Aut–EO and Spr–EO, respectively). This consistent trend across both seasonal EOs reflects a shared steric limitation; the space-restricted DPPH radical system poses a universal barrier to bulky terpene architectures, whereas the planar ABTS radical remains uniformly accessible to both monoterpene and sesquiterpene compounds.

While both seasonal EOs displayed lower antioxidant capacity than gallic acid, their FRSA values remained within the range reported for many commercial EOs (EC_50_ ≈ 0.01–105.3 mg/mL; ascorbic acid EC_50_ = 0.003 mg/mL) [36]. Notably, the BCBA activity of the seasonal EOs (especially Aut–EO) outperformed those of common EOs like cinnamon, lemon balm, cedar, lemon, mandarin, and rosemary (EC_50_ ≈ 0.5–4.8 mg/mL), showing comparable efficacy to thyme EO (EC_50_ = 0.19 mg/mL) and the synthetic antioxidant butylated hydroxyanisole (BHA; EC_50_ = 0.20 mg/mL) [37].

Overall, consistent with the aforementioned antibacterial trends, the quantitative chemical composition variations between Aut–EO and Spr–EO do not seem to be directly reflected in the antioxidant potential of the Az–CJF EO. Although further computational studies would be valuable to clarify the contribution of individual EOCs to the antioxidant properties of this EO, the present results indicate that Az–CJF EO displays interesting potential for use as a BCBA agent in both medicine and the food industry. In fact, the BCBA assay provides a closer model to the real lipid systems occurring in food products and human cells [37]. However, further in vivo evaluation remains necessary to validate its antioxidant efficacy.

#### 2.2.3. Anti-Inflammatory Activity (BSA Denaturation and COX Inhibition Assays)

Due to the established link between oxidative stress and chronic inflammation, EOs with antioxidant potential are often evaluated as promising anti-inflammatory agents [38,39]. In this study, the anti-inflammatory capacity of the seasonal Az–CJF EOs, alongside the non-steroidal anti-inflammatory drug (NSAID) diclofenac sodium as the positive control, was investigated through their ability to inhibit BSA thermal denaturation (Table 4) and COX-1 and COX-2 enzymes (Table 5), with the enzymatic results expressed as half-maximal inhibitory concentration (IC_50_) values.

Regarding the BSA denaturation, both Aut–EO and Spr–EO showed a generally greater protective effect (91–98% and 84–91% inhibition, respectively) than that of the positive control (70–87%), with no statistically significant differences between the two EOs. A two-way analysis of variance (ANOVA) confirmed that the interaction between the seasonal collection period and concentration was not statistically significant, with *F*(4, 20) = 0.055, *p* = 0.994, *η*_p_^2^ = 0.011, indicating that these variables influence the inhibition independently of one another.

Conversely, the COX inhibition assays revealed that Spr-EO was more potent than Aut-EO against both COX-1 (IC_50_: 284 vs. 560 μg/mL) and COX-2 (IC_50_: 750 vs. 1074 μg/mL). Notably, both seasonal EOs displayed a stronger inhibitory effect against COX-1 than COX-2, suggesting a marked selectivity for COX-1. However, diclofenac sodium remained the strongest inhibitor against both enzymes (IC_50_ < 0.2 µg/mL). In natural product research, preferential COX-1 selectivity represents a relevant characteristic for specialized therapeutic designs. Unlike synthetic drugs, multi-component matrices with moderate COX-1 affinity can be useful for targeted applications, such as modulating localized cutaneous inflammation and promoting wound healing, without inducing the systemic or cardiovascular risks often associated with exclusive synthetic COX-2 inhibitors [40,41].

To date, literature reports remain scarce regarding the anti-inflammatory potential of the CJF EO. However, a Korean CJF EO sample was documented to significantly inhibit pro-inflammatory factors, including tumor necrosis factor-α (TNF-α), interleukin-1β (IL-1β), interleukin-6 (IL-6), and nitric oxide (NO), in macrophage models. Furthermore, that study demonstrated that the EO hindered both COX-2 enzyme and corresponding mRNA expression, which correlated with the reported reduction in prostaglandin E_2_ [42]. Interestingly, that EO sample shares a similar qualitative chemical profile with the analyzed Az–CJF EO (Table 1), specifically regarding major EOCs, such as α-pinene (**1**), sabinene (**3**), terpinen-4-ol (**9**), elemol (**12**), eudesmol isomers (**14**–**16**), and kaur-16-ene (**21**). Particularly, a closely matching quantitative chemical profile was observed between Korean EO and Spr–EO samples concerning α-pinene (3.5% vs. 6.5%), total eudesmol isomers content (19.8% vs. 18.6%), and kaur-16-ene (17.2% vs. 23.0%), as well as between Korean EO and Aut–EO samples regarding terpinen-4-ol (4.1% vs. 2.0%) and elemol (10.9% vs. 16.0%). The evaluation of cellular COX-2 expression in the Korean CJF EO and the direct cell-free COX-1 enzymatic binding examined in the current study target distinct physiological mechanisms, thereby providing complementary insights into the anti-inflammatory pathways of these chemically similar CJF EOs. Taken together, these findings confirm that CJF EOs can act as anti-inflammatory agents and serve as sources of EOCs exhibiting this bioactivity.

Overall, in contrast to all the other bioactivities tested, the ability of Az–CJF EO to inhibit COX-1 and COX-2 enzymes is significantly influenced by seasonal variation.

#### 2.2.4. Molecular Docking Insights into COX-1 and COX-2

The anti-inflammatory potential of the seasonal Az–CJF EOs was further investigated through in silico molecular docking studies with COX enzymes. To gain a deeper insight into the molecular mechanisms involved in these anti-inflammatory effects, the EOCs with the most prominent seasonal fluctuations in abundance, including α-pinene (**1**; MH), elemol (**12**; OS), α+β-eudesmol (**15** plus **16**; OS), phyllocladene (**20**; DH), and kaur-16-ene (**21**; DH) (Table 1), were subjected to docking simulations within the active sites of the COX-1 and COX-2 enzymes. The calculated values of binding energy are presented in Table 6.

Regarding COX-1, the predicted binding affinity followed the decreasing order: kaur-16-ene > diclofenac sodium > β-eudesmol > phyllocladene > α-eudesmol > elemol > α-pinene. For the COX-2 isoform, the affinity trend was β-eudesmol = diclofenac sodium > kaur-16-ene > elemol > α-eudesmol = phyllocladene > α-pinene.

**Figure 2:**
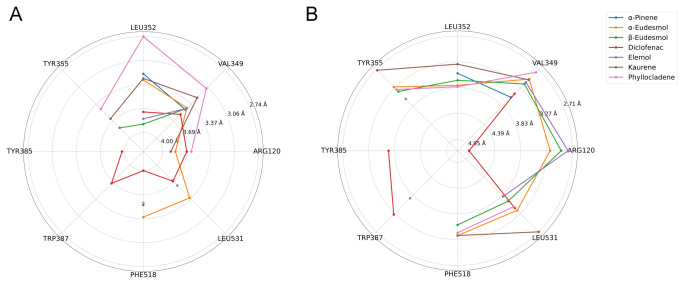
Radar plots representing the interaction distances between the selected ligands and key active-site residues of COX-1 (**A**) and COX-2 (**B**), obtained from Protein–Ligand Interaction Profiler (PLIP) analyses of the best docking poses. Ligands are represented as follows: α-pinene (blue), α-eudesmol (orange), β-eudesmol (green), diclofenac sodium (red), elemol (purple), kaur-16-ene (brown), and phyllocladene (pink). Higher radial values indicate shorter interaction distances and stronger ligand–residue proximity within the cyclooxygenase active site.

Out of all investigated EOCs, kaur-16-ene was predicted to display the highest affinity towards the COX-1 enzyme, with a binding energy of −9.7 kcal/mol. Notably, this value indicated a significantly higher affinity than those obtained for all other studied EOCs and even outperformed the reference NSAID diclofenac sodium (−8.2 kcal/mol). Conversely, for the COX-2 enzyme, the highest affinity was achieved by β-eudesmol and diclofenac sodium, both showing a binding energy of −7.9 kcal/mol, followed by kaur-16-ene (−7.5 kcal/mol). The interaction distances between the ligands and important catalytic pocket residues, such as ARG120, VAL349, LEU352, TYR355, TYR385, TRP387, PHE518, and LEU531, were analyzed using radar plots based on Protein–Ligand Interaction Profiler (PLIP) studies (Figure 2). These residues are well-known to be essential for substrate binding, catalysis, and the binding of inhibitors in cyclooxygenases [40].

In COX-1, diclofenac sodium showed the broadest interaction range among the selected EOCs, interacting with ARG120, VAL349, LEU352, TYR385, TRP387, PHE518, and LEU531. Notably, ARG120, a residue frequently involved in the anchoring of NSAIDs [41], was found to interact with the reference drug, thereby validating the docking protocol. Among the EOCs, kaur-16-ene and phyllocladene were identified as having the most significant interactions within the active site of the COX-1 enzyme. Kaur-16-ene formed hydrophobic interactions with residues including ARG120, VAL349, LEU352, TYR355, and PHE518, while phyllocladene displayed shorter interaction distances to residues LEU352 and VAL349. Although phyllocladene showed shorter interaction distances with these specific residues, it still exhibited a lower binding affinity compared to kaur-16-ene (Figure 2A and Table 6).

Moreover, the eudesmol isomers showed complementary binding affinities to the enzymes. Specifically, α-eudesmol and β-eudesmol interacted with the key residues ARG120, VAL349, LEU352, TYR355, and LEU531 in COX-2, exhibiting an interaction profile more similar to that of diclofenac (Figure 2B). In particular, β-eudesmol showed one of the highest binding energies to COX-2 (−7.9 kcal/mol). These results strongly support the findings regarding the anti-inflammatory properties of eudesmol isomers reported by previous studies [43].

This distinct behavior can be rationalized through a structure-activity relationship (SAR) analysis, which indicates that molecular size, functionalization with oxygenated groups, and enzymatic stereoselectivity are among the primary determinants governing the interaction of selected EOCs with COX enzymes.

The weakest binding affinities were shown by α-pinene due to its small bicyclic hydrocarbon scaffold, which cannot form polar interactions. In contrast, for the OS α-eudesmol, β-eudesmol, and elemol, the binding energies were superior to those of α-pinene due to the presence of hydroxyl groups that stabilize the ligands through the formation of additional polar contact bridges, without a negative effect on the hydrophobic interactions within the binding cyclooxygenase channel.

Nevertheless, kaur-16-ene demonstrated the highest binding affinity toward COX-1, despite lacking oxygenated functionalities. This is likely because its bulky, rigid, and hydrophobic tetracyclic structure presents a remarkable shape complementarity with the hydrophobic pocket of COX-1, perfectly aligning its hydrophobic surface areas efficiently within the binding cyclooxygenase channel. Similar to kaur-16-ene, phyllocladene established some short contacts with a few active-site amino acid residues. However, it demonstrated a lower affinity than kaur-16-ene, implying that ligand binding affinity depends not only on specific distances of interaction but also on the cumulative effect of shape, orientation, and spatial arrangement of the interactions with active site amino acid residues. Indeed, the present in silico results highlight the high stereoselectivity of the COX active site, which efficiently discriminates between the spatial configurations of these two related tetracyclic diterpene isomers.

Therefore, comparing the composition of the seasonal Az–CJF EOs (Table 1) with their docking profiles provided strong evidence to support the hypothesis that the improved anti-inflammatory effect of Spr–EO may be attributed to the substantial 9-fold spike of kaur-16-ene (23.0% compared to 2.6% in Aut–EO), synergizing with a greater abundance of OS, including α- and β-eudesmol.

## 3. Materials and Methods

### 3.1. Chemicals and Reagents

A standard mixture of C7–C33 *n*-alkanes was purchased from Restek (Bellefonte, PA, USA). The COX Colorimetric Inhibitor Screening Assay Kit (Item No. 701050) was purchased from Cayman Chemical^®^ (Ann Arbor, MI, USA). Kanamycin, gallic acid (98%), diclofenac sodium, BSA, DPPH, ABTS, β-carotene, linoleic acid, potassium persulfate (K_2_S_2_O_8_), anhydrous sodium sulfate (Na_2_SO_4_), disodium hydrogen phosphate (Na_2_HPO_4_), sodium dihydrogen phosphate (NaH_2_PO_4_), and sodium chloride (NaCl) were acquired from Sigma-Aldrich (St. Louis, MO, USA). Dichloromethane (≥99.9%), methanol (≥99.8%), ethanol (96%), chloroform (≥99%), and DMSO were obtained from Riedel-de Haën (Seelze, Germany). Nutrient agar (NA), Mueller–Hinton agar (MHA), and lysogeny broth (LB) were supplied by Merck Millipore (Darmstadt, Germany). Deionized water was used throughout all experiments.

### 3.2. Plant Material, Sampling, and Study Area Characterization

The CJF was harvested from a tree population, aged 15–20 years, growing in the Cerrado dos Bezerros Recreational Forest Reserve, located at latitude 37°44′38.347″ N, longitude 25°21′56.263″ W, and an altitude of 400 m, in Vila Franca do Campo on São Miguel Island (Azores, Portugal). The plant material was collected early in the morning during two different periods, namely, November 2022 (autumn) and April 2023 (spring). To mitigate individual biological variability, mixed samples were gathered from multiple representative trees within the population to form a single pooled batch per season. The collected material was immediately brought to the laboratory at the University of the Azores, cleaned, separated from cones, and stored at –20 °C until further HD.

São Miguel belongs to the eastern island group of the Azores archipelago, located in the middle of the Atlantic Ocean. This archipelago, constituted by nine major islands of volcanic origin, is an autonomous region of Portugal, situated roughly 1500 km west of the mainland. Azorean forests are associated with a subtropical climate, characterized by temperate humid conditions with low thermal amplitude all year round, along with mild and relatively wet summers. The climatic parameters, namely, air temperature, precipitation and global solar radiation (GSR) of São Miguel during the sampling months were detailed in Rodrigues et al. [21]. In particular, the average temperature, rainfall and GSR during the months of sample collection were, respectively, 17.7 °C, 71.2 mm and 21,407.5 kJ/m^2^ for November 2022, and 16.8 °C, 66.2 mm and 7243.6 kJ/m^2^ for April 2023.

### 3.3. EOs Extraction Using the HD Process

The obtention of EO from each Az–CJF sample (without cones) was performed via HD, using a Clevenger-type apparatus, according to Rodrigues et al. [21], following European Pharmacopoeia [44] guidelines. Briefly, 300 g of fresh sample, previously cut into smaller pieces (2–3 cm in length), was hydrodistilled with 3 L of water for 3 h using a heating mantle (J.P. Selecta, Abrera, Spain). The distillation time was measured from the appearance of the first drop of distillate. The separated EO was dried over anhydrous Na_2_SO_4_, filtered, weighed, and then stored at 4 °C, in sealed amber vials, for no longer than six months before chemical and bioassay analyses. Each extraction procedure was performed in triplicate and analyzed separately.

### 3.4. EOs Chemical Composition Analysis Through GC–MS

The analyses, following the protocol previously established by the research group [21], were conducted on a Shimadzu GCMS–QP2010 Ultra gas chromatograph–mass spectrometer (Shimadzu Corp., Tokyo, Japan) operating with an electron-impact (EI) ionization source (70 eV). Chromatographic separation was accomplished on a ZB–5MSPlus capillary column (60 m length × 0.25 mm i.d., film thickness 0.25 µm; 5% phenyl, 95% methyl siloxane; Phenomenex Inc., Torrance, CA, USA). Samples (0.1 μL of a 0.1 g/mL solution in dichloromethane) were injected in split mode at a 24.4:1 ratio. Helium was utilized as the carrier gas at a constant flow rate of 36.3 cm/s. The injector and ion source temperatures were maintained at 260 °C. The oven temperature program started at 50 °C, increased to 260 °C at 2 °C/min, and was held isothermally for 5 min. The transfer line temperature was maintained at 300 °C. Mass spectra were acquired over a 40–400 *m*/*z* range with a scan time of 0.3 s.

The retention indices (RI) of the EOCs were calculated relative to a homologous series of *n*-alkanes (C7–C33) and their raw percentages were quantified by integrating the total ion current (TIC) chromatogram peaks.

The identity of the EOCs was assigned by comparison of their RI and mass spectra with those from (i) an in-house library, based upon the analyses of commercially available standards and reference EOs [14] and (ii) commercial libraries (FFNSC4.0, NIST11, and Wiley10). The identity of the EOCs was confirmed through co-injections with available standards.

### 3.5. In Vitro Antibacterial Activity Determination

#### 3.5.1. Bacterial Strains and Suspension Preparation

The bacterial strains tested include four Gram-positive, namely, *Bacillus licheniformis* (Weigmann) Chester (DSM 13), *Bacillus subtilis* (Ehrenberg) Cohn (DSM 10), *Staphylococcus aureus* Rosenbach (DSM 1104) and *Micrococcus luteus* (Schroeter) Cohn (DSM 20030), and three Gram-negative, namely, *Serratia marcescens* Bizio (DSM 48), *Enterobacter cloacae* (Jordan) Hormaeche & Edwards (DSM 30054) and *Escherichia coli* (Migula) Castellani & Chalmers (DSM 498). All bacterial strains were obtained from the Microbiology Laboratory of the Department of Biology, at the University of the Azores, and maintained on solid NA culture medium. For the DDM assay, the bacterial suspensions were prepared by adding the cultured organisms to a 0.9% NaCl solution, adjusting the bacterial concentration to the 0.5 McFarland scale. As for the BMM assay, the suspensions were prepared in LB medium following overnight culture and adjusted with 0.9% NaCl to a final concentration of 10^8^ colony-forming units per milliliter (CFU/mL).

Some of the selected bacterial species are of a particular concern for human health, namely, *S. aureus*, *E. cloacae* and *E. coli*, which belong to the opportunistic ESKAPEE group, constituted by seven bacterial strains with pronounced antibiotic resistance development and highly virulent effects [23]. On the other hand, the selected *Bacillus* spp. represent important food spoilage Bacillales members [45] (e.g., *B. subtilis* is a pathogen of the bakery industry [46]), and have been recently described as opportunistic human pathogens, with *B. licheniformis* being resistant towards penicillin [46]. Concerning the remaining bacteria, *M. luteus* has also been recently considered a potential clinically opportunistic pathogen [47], while *S. marcescens* has recently gained attention as an emerging pathogen worldwide [48].

#### 3.5.2. GIZ Determination via DDM

The antibacterial activity of the EOs was first screened via DDM [49], with some modifications. MHA plates were inoculated with the bacterial suspensions and loaded with 6 mm paper discs containing 5 μL of undiluted EO. Inoculated plates without test samples served as growth controls, while discs with 5 µL of kanamycin (10 mg/mL in water) were utilized as positive control. Plates were incubated for 24 h at 28 °C or 37 °C, for Gram-positive and Gram-negative bacteria, respectively. After incubation, GIZs were measured in mm (including disc diameter). All assays were carried out in triplicate.

#### 3.5.3. MIC Determination via BMM

The antibacterial activity of the EOs was also evaluated using the BMM, according to the CLSI guidelines [50], with slight modifications. Initially, 100 µL of each sample solution (40 mg/mL in 10% DMSO, diluted in the appropriate culture medium) was added to the first well of a 96-well microplate. Two-fold serial dilutions were then performed to yield concentrations from 0.04 to 40 mg/mL. Finally, 100 µL of the bacterial suspension (diluted in the appropriate culture medium) was added to each well to achieve a target density of 10^5^ CFU/mL, resulting in final EO concentrations ranging from 0.02 to 20 mg/mL. Bacterial growth controls (including 10% DMSO) and culture medium sterility controls were included, while kanamycin was utilized as the positive control. The prepared microplates were then sealed with sterile covers and parafilm, and incubated for 24 h at 28 °C or 37 °C for Gram-positive and Gram-negative bacteria, respectively. The MIC values were determined by measuring the absorbance (Abs) at 596 nm using a microplate reader (Thermo Scientific Multiskan FC, Waltham, MA, USA). All assays were conducted in triplicate.

### 3.6. In Vitro Antioxidant Activity Determination

The antioxidant activity of the EOs, alongside the reference antioxidant gallic acid as the positive control, was estimated using three chemical-based assays. Specifically, two FRSA methods were employed, namely the DPPH and ABTS tests, both based on mixed HAT/SET reactions [35]. Additionally, a lipid peroxidation assay, the BCBA (a HAT-based method [35]) was performed. All assays were adapted from the modified protocols described by Lima et al. [51] and carried out in triplicate, with results expressed as EC_50_ values. To ensure optimal homogeneity and solubility between the hydrophobic EOs and the polar reaction systems, absolute methanol and ethanol were strictly utilized as mutual solubilizing solvents across all chemical assays.

#### 3.6.1. DPPH–FRSA Assay

Briefly, a 100 μL aliquot of each sample solution (0.15–150 mg/mL in methanol) was mixed in a 96-well microplate with 100 μL of methanolic DPPH solution. The mixture was then incubated in the dark for 30 min at room temperature, and its Abs measured at 520 nm using a microplate reader, against a blank solution containing all reagents except the test sample. The scavenging effect percentage was calculated using the following equation: DPPH–FRSA (%) = [(Abs Blank − Abs Sample)/Abs Blank] × 100. The EC_50_ value (mg/mL) was defined as the sample concentration required to scavenge 50% of the DPPH radicals.

#### 3.6.2. ABTS–FRSA Assay

Briefly, a 100 μL aliquot of each sample solution (0.15–150 mg/mL in methanol) was mixed in a 96-well microplate with 100 μL of ABTS solution (prepared as previously reported [51]). The mixture was then incubated in the dark for 10 min at room temperature, and its Abs measured at 734 nm using a microplate reader, against a blank solution containing all reagents except the test sample. The scavenging effect percentage was calculated using the following equation: ABTS–FRSA (%) = [(Abs Blank − Abs Sample)/Abs Blank] × 100. The EC_50_ value (mg/mL) was defined as the sample concentration required to scavenge 50% of the ABTS radicals.

#### 3.6.3. BCBA Assay

Briefly, a 50 μL aliquot of each sample solution (0.012–6.25 mg/mL in ethanol) was mixed in a 96-well microplate with 250 μL of β-carotene-linoleic acid emulsion (prepared as previously reported [51]). Immediately after mixing (t = 0), the initial Abs was measured at 450 nm using a microplate reader. The mixture was then incubated for 180 min at 52 °C, and the final Abs (t = 180) was recorded. A blank without β-carotene was prepared for background subtraction, while the control contained 50 μL of deionized water instead of the sample. The BCBA was calculated as percent inhibition relative to the control using the following equation: BCBA (%) = [(Abs Sample − Abs Control) _t=180 min_/(Abs Control _t=0_ − Abs Control _t=180 min_)] × 100. The EC_50_ value (mg/mL) was defined as the sample concentration required to achieve 50% inhibition of the β-carotene bleaching.

### 3.7. In Vitro Anti-Inflammatory Activity Determination

The anti-inflammatory activity of the EOs, alongside the NSAID diclofenac sodium as the positive control, was estimated through their ability to inhibit BSA denaturation and COX isoforms (COX-1/COX-2).

#### 3.7.1. BSA Denaturation Inhibition Assay

BSA denaturation inhibition was assessed according to Matotoka et al. [52], with slight modifications for a microplate format [53]. Stock solutions (100 mg/mL in DMSO) were diluted in phosphate-buffered saline (PBS, pH 6.4) to obtain a working range of 0.0011–0.1417 mg/mL. The reaction mixtures (300 μL) consisted of 50 μL of each sample and 250 μL of a 4% BSA solution. To account for EO coloration, sample blanks were prepared by mixing 50 μL of each sample solution with 250 μL of PBS. A control solution, representing maximum protein denaturation, contained 250 μL of 4% BSA and 50 μL of PBS. All mixtures were heated at 90 °C for 14 min to induce protein denaturation, and then cooled to room temperature. Turbidity was measured at 650 nm using a microplate reader against a PBS blank. All assays were performed in triplicate, and the protein denaturation inhibition percentage was calculated using the following equation: Inhibition (%) = [(Abs Control − (Abs Sample − Abs Sample Blank))/Abs Control] × 100.

#### 3.7.2. COX-1 and COX-2 Inhibition Assay

The inhibitory effects of the EOs on COX-1 and COX-2 enzymes were evaluated using the previously described commercial screening kit (Section 3.1), according to the manufacturer’s instructions. Briefly, 150 µL of assay buffer, 10 µL of hemin, 10 µL of COX-1 enzyme, and 10 µL of each EO (4–2000 µg/mL) or diclofenac sodium (0.2–100 µg/mL, as the positive control) were added to each well of a 96-well plate and incubated at 25 °C for 5 min. Subsequently, 20 µL of the colorimetric substrate solution and 20 µL of arachidonic acid were added to initiate the reaction. The plate was incubated at 25 °C for an additional 2 min, and the Abs was measured at 590 nm using a microplate reader. A similar procedure was carried out for the COX-2 inhibition assay.

### 3.8. Molecular Docking Studies on COX-1 and COX-2

The major EOCs with the highest variation yields between Aut–EO and Spr–EO, such as kaur-16-ene, phyllocladene, α-eudesmol, β-eudesmol, elemol, and the α-pinene, were selected for molecular docking studies to investigate their potential interactions with inflammatory targets, specifically COX isoforms (COX-1 and COX-2). Diclofenac sodium was used as a reference compound. The structures of COX-1 (7JXT) and COX-2 (5KIR) enzymes were retrieved from the Protein Data Bank (PDB), and were prepared in AutoDockTools version 1.5.6 [54] for repairing missing atoms, adding hydrogen atoms, assigning Kollman charges, and determining the state of His residues. The grid box was defined by selecting the ligand of each enzyme to capture the entire pocket and optimize the docking site. Before docking simulations, the structures without ligands were subjected to energy minimization using the optimized potentials for liquid simulations (OPLS) force field using GROMACS 2026.2 [55]. Ligand structures were obtained from the PubChem database, converted to PDB using the PyMOL (The PyMOL Molecular Graphics System, Version 2.3.3 Schrödinger, LLC, Portland, OR, USA) and then prepared using AutoDockTools to assign Gasteiger charges and detect rotatable bonds. Subsequently, molecular docking simulations were carried out using the AutoDock Vina software 1.1.2. version [56]. The PLIP web tool [57] was employed to analyze all types of interactions between the docked ligands and the COX enzymes. Docking poses were selected according to both binding affinity scores and the quality of interactions established with key residues within the enzyme active site. Radar plots illustrating the interaction distances between ligands and active-site residues of COX-1 and COX-2 were generated from PLIP output files using Python 3 (version 3.10.0) and the Matplotlib package (version 3.7.1).

### 3.9. Statistical Analysis

Data were expressed as mean ± standard deviation (SD) of at least three independent experiments. Statistical analysis was conducted using IBM SPSS Statistics version 29.0.2.0 software (SPSS Inc., Chicago, IL, USA). The normal distribution of continuous variables was verified using the Shapiro–Wilk test. A paired-samples *t*-test was used to analyze differences in the chemical composition and bioactivities of the EOs between the autumn and spring seasons. One-way ANOVA followed by Duncan’s multiple-range test was performed to determine statistical differences among the samples and standards in the antibacterial, antioxidant and anti-inflammatory assays. Additionally, a two-way ANOVA was applied to evaluate potential interaction factors between independent variables in the BSA denaturation inhibition assay. Correlations among the antioxidant activity methods were assessed using Pearson’s correlation coefficient (*r*). Differences were considered statistically significant at a 5% significant level (*p* < 0.05).

## 4. Conclusions

In conclusion, this study demonstrates that the core antibacterial, antioxidant, and BSA denaturation inhibitory activities of Az–CJF EO remain stable regardless of the harvesting season (autumn vs. spring), thereby confirming that seasonal fluctuations in individual constituents do not disrupt the baseline chemical homeostasis and overall bioactive potential of this valorized biomass. Crucially, however, the anti-inflammatory activity via the COX-1/COX-2 pathways proved to be seasonally dependent, with the Spr–EO exhibiting significantly higher inhibition. Molecular docking trends support these findings, suggesting that this enhanced effect may be driven by the seasonal enrichment of the DH kaur-16-ene and OS, such as α- and β-eudesmol.

When contextualized globally, the bioactivity profile of Az–CJF EO aligns with the established consensus that CJF chemotype EOs consistently share a baseline antibacterial efficacy. Additionally, by reporting further data on its potential lipid-protective capacity and targeted COX-1 selectivity (aspects less documented for CJF EOs), this study contributes to a more comprehensive pharmacological fingerprint for the Az–CJF EO, which may be influenced by the continuous abiotic pressures of the region’s hyperhumid insular climate.

Although, with the exception of the BSA denaturation inhibition, these bioactivity potencies are generally weak to moderate compared to commercial reference drugs; under the experimental conditions employed, they fall well within established thresholds for crude EOs. Consequently, this comparative study establishes these seasonal variations as fundamental quality assurance parameters for future batch standardization. By merging these stable core activities and seasonal anti-inflammatory profiles with previously established anticholinergic findings, Az–CJF EO shows promise as a natural MTDL for standardized therapeutic applications within a circular bioeconomy.

Nevertheless, advanced formulation strategies, such as nanoencapsulation or topical emulsions, are recommended to circumvent the physicochemical limitations of the crude EO, ensuring a safer and more efficient delivery. Further in vivo evaluations and clinical trials remain necessary to fully validate the translational potential of Az–CJF EO in managing inflammatory and oxidative stress-related disorders.

## Figures and Tables

**Table 1:** Major components (≥1% total peak area) and yield (%) of the essential oil (EO) from Azorean *Cryptomeria japonica* foliage harvested in autumn (Aut) and spring (Spr) (adapted from Rodrigues et al. [21]).

Major Components *					Aut–EO	Spr–EO	Aut–EO	Spr–EO
No.	Class	Name	RI_C_	RI_L_	Relative Content (%)		Seasonal Proportion	
1	MH	**α-Pinene**	929	932	**11.2 ± 1.0 ^a^**	**6.5 ± 0.7 ^b^**	**1.7 ↑**	
2	MH	Camphene	945	946	1.5 ± 0.2 ^a^	1.6 ± 0.3 ^a^		1.1 ↑
3	MH	**Sabinene**	967	969	**8.1 ± 1.6 ^a^**	**1.6 ± 0.2 ^b^**	**5.1 ↑**	
4	MH	β-Pinene	973	974	0.8 ± 0.04 ^a^	0.5 ± 0.03 ^a^	1.6 ↑	
5	MH	β-Myrcene	984	988	2.4 ± 0.3 ^a^	1.5 ± 0.2 ^b^	1.6 ↑	
6	MH	δ-3-Carene	1005	1008	0.3 ± 0.03 ^b^	1.0 ± 0.1 ^a^	-	3.3 ↑
7	MH	**Limonene**	1025	1024	**7.4 ± 2.2 ^a^**	**2.0 ± 0.3 ^b^**	**3.7 ↑**	
8	MH	γ-Terpinene	1053	1054	0.9 ± 0.4 ^a^	0.2 ± 0.1 ^b^	4.5 ↑	
9	OM	Terpinen-4-ol	1175	1174	2.0 ± 0.7 ^a^	0.6 ± 0.1 ^b^	3.3 ↑	
10	OM	Bornyl acetate	1278	1287	1.6 ± 0.2 ^b^	2.5 ± 0.2 ^a^		1.6 ↑
11	SH	δ-Cadinene	1510	1514	0.9 ± 0.04 ^a^	1.0 ± 0.05 ^a^		1.1 ↑
12	OS	**Elemol**	1541	1548	**16.0 ± 2.3 ^b^**	**20.8 ± 1.6 ^a^**		**1.3 ↑**
13	OS	Germacrene D-4-ol	1568	1574	1.0 ± 0.0 ^a^	1.3 ± 0.0 ^a^		1.3 ↑
14	OS	**γ-Eudesmol**	1623	1630	**3.9 ± 0.3 ^b^**	**6.0 ± 0.5 ^a^**		**1.5 ↑**
15/16	OS	**α+β-Eudesmol**	1645	1649/1652	**7.8 ± 0.5 ^b^**	**12.6 ± 0.8 ^a^**		**1.6 ↑**
17	DH	Rosa-5,15-diene	1921	1926	0.8 ± 0.1 ^a^	0.03 ± 0.00 ^b^	27.0 ↑	
18	DH	Kryptomeren	1924	1933	0.9 ± 0.1 ^a^	0.3 ± 0.06 ^b^	3.0 ↑	
19	DH	Pimaradiene	1935	1948	1.2 ± 0.2 ^a^	0.3 ± 0.07 ^b^	4.0 ↑	
20	DH	**Phyllocladene**	2011	2016	**18.9 ± 2.0 ^a^**	**5.1 ± 0.6 ^b^**	**3.7 ↑**	
21	DH	**Kaur-16-ene**	2034	2042	**2.6 ± 0.2 ^b^**	**23.0 ± 2.1 ^a^**		**8.8 ↑**
22	OD	Nezukol	2120	2132	1.6 ± 0.5 ^a^	2.7 ± 0.4 ^a^		1.7 ↑
Total grouped components (%)								
Monoterpenes hydrocarbons (MH)					35.0 ± 6.1 ^a^	15.8 ± 2.1 ^b^	2.2 ↑	
Oxygenated monoterpenes (OM)					4.5 ± 1.0 ^a^	3.9 ± 0.4 ^a^	1.1 ↑	
Sesquiterpenes hydrocarbons (SH)					1.6 ± 0.02 ^a^	2.2 ± 0.2 ^a^		1.4 ↑
Oxygenated sesquiterpenes (OS)					30.7 ± 4.0 ^b^	44.6 ± 3.2 ^a^		1.4 ↑
Diterpenes hydrocarbons (DH)					25.5 ± 2.2 ^a^	29.3 ± 3.0 ^a^		1.1 ↑
Oxygenated diterpenes (OD)					1.7 ± 0.6 ^a^	2.8 ± 0.4 ^a^		1.6 ↑
Total identified components (%)					99.0 ± 0.4 ^a^	98.6 ± 0.6 ^a^		
Total terpenes (%)					62.1 ± 3.9 ^a^	47.3 ± 3.9 ^b^	1.3 ↑	
Total terpenoids (%)					36.9 ± 3.5 ^b^	51.3 ± 2.2 ^a^		1.4 ↑
Terpene-to-terpenoid ratio					1.7 ± 0.3 ^a^	0.9 ± 0.1 ^b^	1.9 ↑	
**EO yield (%, *v*/*w*, dry matter)**					1.4 ± 0.2 ^a^	1.1 ± 0.07 ^a^	1.3 ↑	

Results are expressed as mean ± SD (*n* = 3). Different superscript letters in the same row (a,b) indicate statistically significant differences at *p* < 0.05. Components higher than 5% are highlighted in boldface. RI_C,_ retention indices calculated relative to C7–C33 *n*-alkanes on a ZB–5MSPlus capillary column; RI_L_, retention indices from the literature [21]. * The identity of all major components, except **18**, was confirmed through co-injections with authentic samples. The superscript arrow (↑) indicates the raw fold-change ratio between the seasonal EOs.

**Table 2:** Antibacterial activity (GIZ and MIC values) of the essential oil (EO) from Azorean *Cryptomeria japonica* foliage harvested in autumn (Aut) and spring (Spr).

Bacterial Strains	Samples/Antibacterial Activity					
	GIZ (mm)			MIC (mg/mL)		
	Aut–EO	Spr–EO	Kanamycin	Aut–EO	Spr–EO	Kanamycin
**Gram-Positive**						
*Bacillus licheniformis*	7.8 ± 0.3 ^b^	8.1 ± 0.1 ^b^	29.3 ± 1.5 ^a^	10.0	>20.0	<0.001
*Bacillus subtilis*	7.0 ± 0.0 ^b^	6.7 ± 0.3 ^b^	27.5 ± 2.1 ^a^	10.0	10.0	<0.001
*Staphylococcus aureus*	6.8 ± 0.3 ^b^	6.7 ± 0.3 ^b^	31.0 ± 3.6 ^a^	20.0	>20.0	<0.001
*Micrococcus luteus*	6.1 ± 0.1 ^b^	6.1 ± 0.0 ^b^	25.7 ± 0.6 ^a^	5.0	5.0	<0.001
**Gram-Negative**						
*Escherichia coli*	na	na	24.7 ± 0.6 ^a^	>20.0	>20.0	<0.001
*Serratia marcescens*	na	na	26.3 ± 0.6 ^a^	>20.0	>20.0	<0.001
*Enterobacter cloacae*	na	na	25.7 ± 0.6 ^a^	>20.0	>20.0	<0.001

Results are expressed as mean ± SD (*n* = 3). Different superscript letters (a,b) in the same row within the GIZ parameters indicate statistically significant differences at *p* < 0.05. Antibacterial activity criteria (GIZ values): not active (≤6 mm); weak (>6–10 mm); moderate (>10–15 mm); strong (>15 mm). GIZ, growth inhibition zone; MIC, minimum inhibitory concentration; na, no activity.

**Table 3:** Antioxidant activity (EC_50_ values) of the essential oil (EO) from Azorean *Cryptomeria japonica* foliage harvested in autumn (Aut) and spring (Spr).

Samples	Antioxidant Activity, EC_50_ (mg/mL)		
	DPPH–FRSA	ABTS–FRSA	BCBA
Aut–EO	10.5 ± 2.0 ^b^	5.6 ± 0.2 ^b^	0.2 ± 0.02 ^b^
Spr–EO	8.2 ± 0.6 ^b^	6.0 ± 0.2 ^b^	0.3 ± 0.02 ^b^
Gallic acid	0.002 ± 0.001 ^a^	0.001 ± 0.001 ^a^	0.02 ± 0.02 ^a^

Results are expressed as mean ± SD (*n* = 3). Different superscript letters in the same column (a,b) indicate statistically significant differences at *p* < 0.05. FRSA, free radical scavenging activity; DPPH, 2,2-diphenyl-1-picrylhydrazyl; ABTS, 2,2′-azino-bis(3-ethylbenzothiazoline-6-sulfonic acid); BCBA, β-carotene-linoleic acid bleaching activity.

**Table 4:** Protective effect of the essential oil (EO) from Azorean *Cryptomeria japonica* foliage harvested in autumn (Aut) and spring (Spr) against bovine serum albumin (BSA) denaturation.

Samples	BSA Denaturation Inhibition (%)				
	2.2 μg/mL	4.4 μg/mL	8.8 μg/mL	17.6 μg/mL	35.2 μg/mL
Aut–EO	91 ± 8 ^a^	97 ± 2 ^a^	98 ± 2 ^a^	98 ± 1 ^a^	98 ± 2 ^a^
Spr–EO	84 ± 13 ^ab^	90 ± 12 ^a^	91 ± 12 ^a^	91 ± 14 ^a^	91 ± 14 ^a^
Diclofenac sodium	70 ± 7 ^b^	80 ± 6 ^b^	84 ± 7 ^b^	87 ± 8 ^b^	87 ± 10 ^b^

Results are expressed as mean ± SD (*n* = 3). Different superscript letters in the same column (a,ab,b) indicate statistically significant differences at *p* < 0.05.

**Table 5:** COX-1 and COX-2 inhibition of the essential oil (EO) from Azorean *Cryptomeria japonica* foliage harvested in autumn (Aut) and spring (Spr).

Samples	Anti-Cyclooxygenases Activity (IC_50_, μg/mL)	
	COX-1	COX-2
Aut–EO	560 ± 87 ^c^	1074 ± 250 ^c^
Spr–EO	284 ± 44 ^b^	750 ± 102 ^b^
Diclofenac Sodium	<0.2 ^a^	<0.2 ^a^

Results are expressed as mean ± SD (*n* = 3). Different superscript letters in the same column (a,b,c) indicate statistically significant differences at *p* < 0.05. COX, cyclooxygenase.

**Table 6:** Best docking models and binding affinity scores of selected components of the essential oil from Azorean *Cryptomeria japonica* foliage and diclofenac sodium against the COX-1 and COX-2 enzymes, obtained by molecular docking analysis using AutoDock Vina.

Ligand	Best Model (COX-1)	COX-1 Affinity (kcal/mol)	Best Model (COX-2)	COX-2 Affinity (kcal/mol)
α-Pinene	10	−5.5	1	−6.3
Elemol	2	−7.2	1	−7.2
α-Eudesmol	6	−7.5	3	−7.1
β-Eudesmol	2	−7.8	1	−7.9
Phyllocladene	9	−7.6	4	−7.1
Kaur-16-ene	1	−9.7	3	−7.5
Diclofenac sodium	1	−8.2	5	−7.9

COX-1, cyclooxygenase-1; COX-2, cyclooxygenase-2.

## Data Availability

Data are contained within the article.

## Abbreviations

Abs, absorbance; ABTS, 2,2′-azino-bis(3-ethylbenzothiazoline-6-sulfonic acid); ANOVA, analysis of variance; Aut, autumn; Az, Azorean; BCBA, β-carotene-linoleic acid bleaching activity; BHA, butylated hydroxyanisole; BMM, broth microdilution method; BSA, bovine serum albumin; CJF, *Cryptomeria japonica* foliage; COX, cyclooxygenase; DDM, disc diffusion method; DH, diterpene hydrocarbons; DPPH, 2,2-diphenyl-1-picrylhydrazyl; EC_50_, half-maximal effective concentration; EO, essential oil; EOC, essential oil component; FRSA, free radical scavenging activity; GC–MS, gas chromatography–mass spectrometry; GIZ, growth inhibition zone; GSR, global solar radiation; HAT, hydrogen atom transfer; HD, hydrodistillation; IC_50_, half-maximal inhibitory concentration; LB, lysogeny broth; LPS, lipopolysaccharide; MH, monoterpene hydrocarbons; MHA, Mueller–Hinton agar; MIC, minimum inhibitory concentration; MTDL, multi-target directed ligand; NA, nutrient agar; NSAID, non-steroidal anti-inflammatory drug; OD, oxygenated diterpenes; OM, oxygenated monoterpenes; OS, oxygenated sesquiterpenes; PDB, Protein Data Bank; RI, retention index; SD, standard deviation; PLIP, Protein–Ligand Interaction Profiler; PSMs, plant secondary metabolites; SAR, structure-activity relationship; SET, single electron transfer; SH, sesquiterpene hydrocarbons; Spr, spring.
